# Induction of Mitochondrial Dependent Apoptosis in Human Leukemia K562 Cells by *Meconopsis integrifolia*: A Species from Traditional Tibetan Medicine

**DOI:** 10.3390/molecules200711981

**Published:** 2015-06-30

**Authors:** Jianping Fan, Pan Wang, Xiaobing Wang, Wei Tang, Chunliang Liu, Yaqin Wang, Wenjuan Yuan, Lulu Kong, Quanhong Liu

**Affiliations:** 1 Co-Innovation Center for Qinba Regions’ Sustainable Development, College of Life Sciences, Shaanxi Normal University, No. 620, West Chang’an Avenue, Chang’an District, 710119 Xi’an, China; E-Mails: qhfanjianping@126.com (J.F.); wangpan@snnu.edu.cn (P.W.); wangxiaobing@snnu.edu.cn (X.W.); tangwei@snnu.edu.cn (W.T.); 41208166@snnu.edu.cn (C.L.); wangyaqinjilin@snnu.edu.cn (Y.W.); yuanwenjuan@snnu.edu.dn (W.Y.); konglulu@snnu.edu.cn (L.K.); 2 College of Life Sciences, Qinghai Normal University, No.38, West Wusi Road, 810008 Xining, China

**Keywords:** *M. integrifolia* ethanol extract, K562 cells, apoptosis, mitochondria, G2**/**M phase arrest

## Abstract

Objectives: *Meconopsis integrifolia* (*M. integrifolia*) is one of the most popular members in *Traditional Tibetan Medicine*. This study aimed to investigate the anticancer effect of *M. integrifolia* and to detect the underlying mechanisms of these effects. Methods: 3-(4,5-dimethylthiazol-2-yl)-2,5-diphenyl tetrazolium bromide (MTT) assay and trypan blue assay were used to evaluate the cytotoxicity of *M. integrifolia*. Changes in cell nuclear morphology and reactive oxygen species (ROS) level were observed by fluorescent microscopy. Apoptosis ratio, DNA damage and mitochondrial membrane potential (MMP) loss were analyzed by flow cytometry. Western blotting assay was adopted to detect the proteins related to apoptosis. Immunofluorescence was used to observe the release of cytochrome C. Results: The obtained data revealed that *M. integrifolia* could significantly inhibit K562 cell viability, mainly by targeting apoptosis induction and cell cycle arrest in G2/M phase. Collapse in cell morphology, chromatin condensation, DNA damage and ROS accumulation were observed. Further mechanism detection revealed that mitochondrion might be a key factor in *M. integrifolia*-induced apoptosis. Conclusions: *M. integrifolia* could induce mitochondria mediated apoptosis and cell cycle arrest in G2/M phase with little damage to normal cells, suggesting that *M. integrifolia* might be a potential and efficient anticancer agent that deserves further investigation.

## 1. Introduction

Tibetan medicine is one of the most important ethnic drugs in traditional Chinese medicine and has been popular worldwide, especially in India and Europe. Its usage has grown significantly in recent years because of the trend of international movement towards natural products. Tibetan medical herbs grow in extreme alpine environments that include high altitude, hypothermic and hypoxic conditions, considerable temperature ranges and strong exposure to sun and ultraviolet radiation, which makes these medical plants more effective, especially on cardiovascular and rheumatism diseases. The genus *Papaver* *(Papaveraceae)*, one of the most popular stars in Tibetan medicine, comprises approximately 80 types of annual, biennial and perennial herbs, mainly distributed in Central and Southwestern Asia, Central and Southern Europe and North Africa [1,2]. *Meconopsis*, an endangered genus of ornamental flowers, belongs to the *Papaveraceae* family. It has been used to treat multiple diseases for hundreds of years in Tibet [3]. Genus *Meconopsis* comprises approximately 50 species, wherein, 43 species are mainly distributed in the Qinghai-Tibetan Plateau (QPT) and the neighboring mountains, except for a few that inhabit in Europe [4,5]. *Meconopsis* species have been increasingly attractive for scholars not only because of their beautiful flowers but also their medicinal functions. Some *Meconopsis* species have been reported to have anti-inflammatory and antioxidant effects [6,7]. For example, *Meconopsis integrifolia* *(M. integrifolia) Franch*, belonging to the *Meconopsis* species, was firstly reported to treat hepatitis, pneumonia, and edema in the eighth century. *M. integrifolia* is a flagship species of the alpine scree in the *Qinghai–Tibetan Plateau*. The bright yellow flowers and leaf blade margins of the species make it distinguishable from others [5].

Modern pharmacological research on *M. integrifolia* has mainly focused on the hepatoprotective effects in rats. G. Zhou has proved that ethanol extract of *Meconopsis* *integrifolia* exhibited excellent hepatoprotective effects *in vitro* and antioxidant activity in rats with CCl_4_-induced liver injury *in vivo* [8]. To our best knowledge, this research uniquely addresses its biological function. Furthermore, it has been reported that alkaloids, flavonoids, and phenylpropanoids are abundant in the *Meconopsis* species including *M. integrifolia* [9]. Studies have also shown that flavonoids are relatively high in *M. integrifolia* [10]. It is well established that many flavonoids have outstanding anticancer effects. For example, studies revealed that quercetin could induce apoptosis in tumor cells [11,12]. Taken together, in this study, we chose K562 cells, which have been reported to be quite resistant to conventional anti-tumor drugs, as the subject to study the tumor inhibition activity of *M. integrifolia* extract. Interestingly, our results showed that K562 cells displayed a relatively high sensitivity to *M. integrifolia*. Our data indicated that *M. integrifolia* could induce K562 cell apoptosis and cell cycle arrest in G2/M phase. In addition, the role of mitochondria in *M. integrifolia-* induced apoptosis was elucidated in this study.

## 2. Results and Discussion

### 2.1. Cytotoxicity of M. integrifolia

In this paper, we first investigated the *in vitro* effects of *M. integrifolia* on the apoptotic cascade in K562 cells. After incubation with *M. integrifolia*, the cells were analyzed for their proliferation rate, nuclear alteration, cell cycle distribution, caspase activity and mitochondrial damage with the goal of elucidating the underlying mechanism of *M. integrifolia* induced-apoptosis.

**Figure 1:**
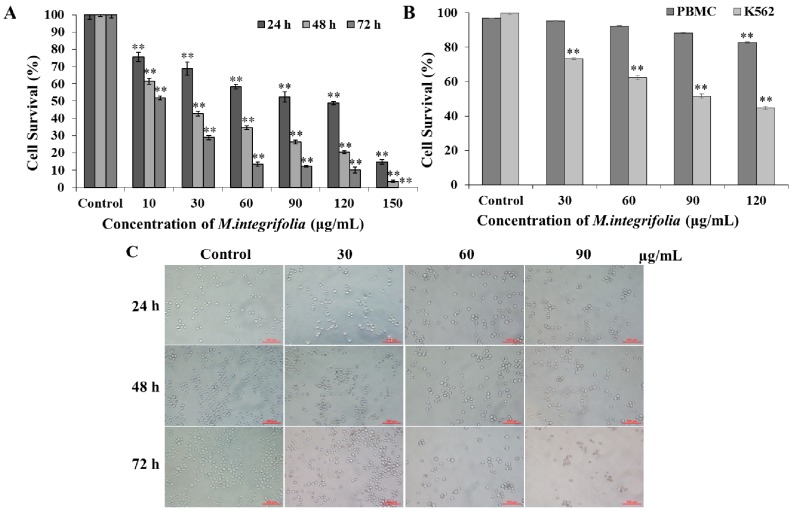
Cell viability tests. (**A**) K562 cells viability after *M. integrifolia* treatment was measured by MTT assay; (**B**) Cytotoxicity effect of *M. integrifolia* on PBMCs and K562 cells through Typan blue assay; (**C**) After treatment with different concentrations of *M. integrifolia* for 24, 48 and 72 h, cell morphology was observed by a phase-contrast microscopy. Each value was expressed as a mean ± S.D. of at least three independent determinations. One-way ANOVA was used for comparisons of multiple group means followed by Dunnett’s *t*-test. ** *p* < 0.01 *vs.* Control. (Error bars = S.D., *n* = 3).

We measured K562 cell viability when treated with various doses of *M. integrifolia* (0, 10, 30, 60, 90, 120 and 150 μg/mL) at cell densities of 1 × 10^5^ cells/mL. According to Figure 1A, *M. integrifolia* inhibited K562 cell proliferation in a dose- and incubation time-dependent manner. The IC_50_ values were calculated as 62.07, 19.62 and 10.85 μg/mL at 24, 48 and 72 h, respectively.

For comparison, Figure 1B reflected the cytotoxicity of *M. integrifolia* on human peripheral blood mononuclear cells (PBMCs), much weaker cell damage was observed in PBMCs compared with cancer cells under the same dose of *M. integrifolia*, suggesting *M. integrifolia* has some selective tumor cell killing effect.

According to the cell viability test, three doses of *M. integrifolia* (0, 30, 60 and 90 μg/mL) were chosen for subsequent experiments in this study. To further confirm the data from cytotoxicity evaluation, alterations in cell number after *M. integrifolia* extract incubation were observed. As shown in Figure 1C, more serious decrease in cell number after *M. integrifolia* treatment was observed as the dose of the extract and incubation time increased.

The data presented above demonstrated that *M. integrifolia* could significantly decrease K562 cell viability as the drug dose and incubation time increased without damaging the normal cells.

### 2.2. M. integrifolia Causes Cell Cycle Arrest in K562 Cells

**Figure 2:**
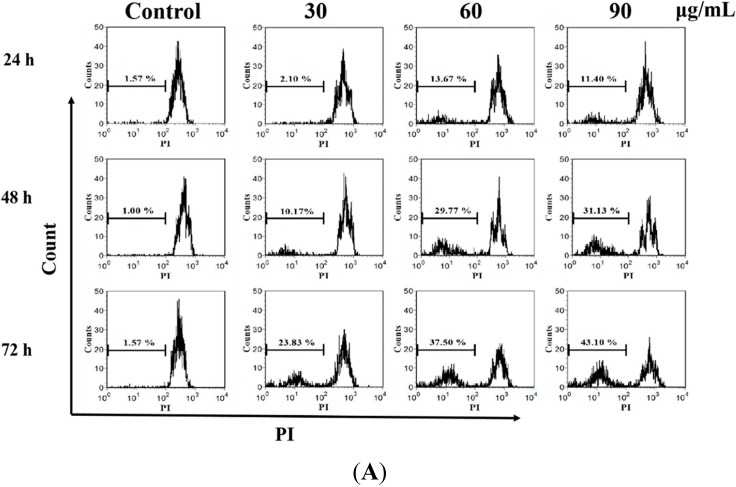

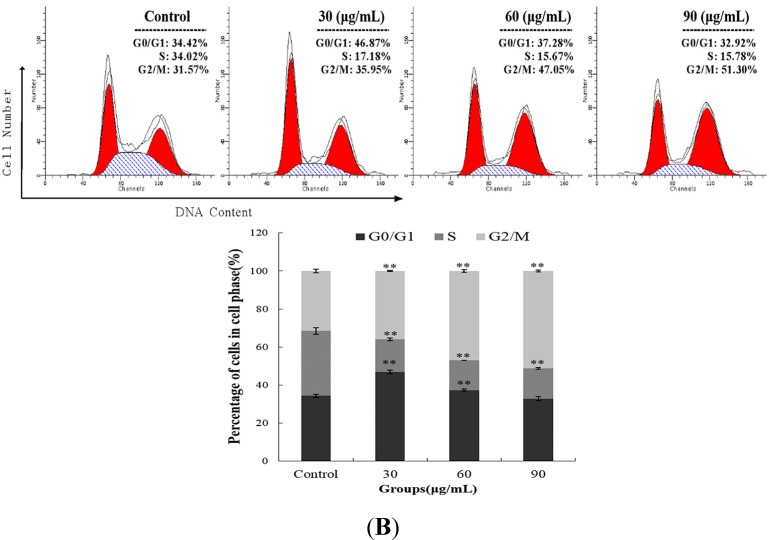
DNA damage and cell cycle arrest post *M. integrifolia* treatment. (**A**) Effect of *M. integrifolia* on DNA fragmentation of K562 cells. K562 cells were treated with *M. integrifolia* for 24, 48 and 72 h, then stained with propidium iodide (PI) and analyzed by flow cytometry; (**B**) Cell cycle analysis of *M. integrifolia*-treated cells. K562 cells were harvested and fixed in 70% alcohol and then stained with PI. Finally the stained cells were analyzed using a flow cytometer. ** *p* < 0.01 *vs.* Control. (Error bars = S.D., *n* = 3).

DNA damage causes genomic instability and ultimately may lead to cancer. Paradoxically, induction of DNA damage, such as DNA double-strand break (DSB), has been shown to be an effective cancer treatment [13]. *Yukyung Karne*, a traditional Tibetan medicine was able to cause DNA fragmentation and induce cell cycle arrest in many cancer cells [14]. Thus, DNA damage was evaluated in the present study. As shown in Figure 2A, we found that cells treated by *M. integrifolia* extract displayed an increase in DNA damage in a dose- and time-dependent manner. *M. Integrifolia* at 90 μg/mL triggered a 7.32-fold increase in DNA damage in K562 cells after 24 h treatment, a 27.45-fold (*p* < 0.01) increase for 48 h, and a 31.13-fold (*p* < 0.01) increase for 72 h. Moreover, preventing tumorigenesis often involves signal transduction pathway modulation, resulting in cell cycle arrest and, eventually, apoptosis [15,16,17]. Therefore, cell-cycle analyses were performed to determine whether the suppression of the proliferative effect of *M. integrifolia* was associated with uncoupling of the cell-cycle progression profile. The data presented in Figure 2B demonstrate that *M. integrifolia* can arrest K562 cells in G2/M phase. After 90 μg/mL *M. Integrifolia* treatment for 24 h, the accumulation of K562 cells in the G2/M phase increased from 30.98% to 51.58% (*p* < 0.01), while the proportion of cells in G1 phase decreased from 35.36% to 32.34% and the proportion of cells in S phase were decreased from 32.01% to 16.05% (*p* < 0.01).

### 2.3. M. integrifolia Triggers Cell Apoptosis in K562 Cells

**Figure 3:**
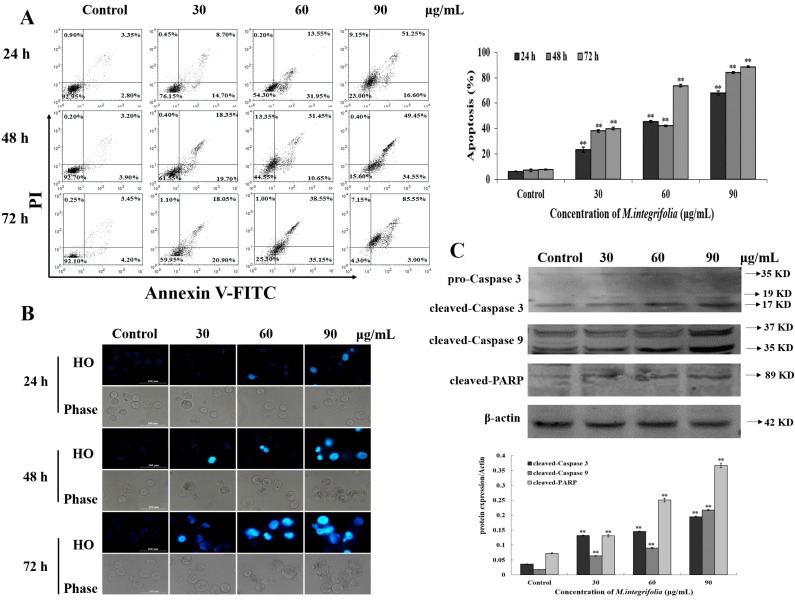
Apoptosis detection in *M. integrifolia*-treated cells. (**A**) The quantification of apoptotic cells. K562 Cells were double-stained with Annexin V-FITC and PI, and then analyzed by flow cytometry; (**B**) Effects of *M. integrifolia* on cell morphology and nucleus of K562 cells. Cells treated for 24, 48 and 72 h were stained with Hoechst 33342. Morphological changes were observed under fluorescent microscope. All experiments were done independently in triplicate per experimental point, and representative results were shown. The results represented the mean ± S.D. of three independent experiments. ** *p* < 0.01 indicated statistically significant differences *vs.* Control; (**C**) Effects of *M. integrifolia* on the expression of some key apoptotic proteins in K562 cells. K562 cells were treated with *M. integrifolia* for 24 h. Western blot analysis was performed in triplicate per experimental point; β-actin was used as reference control.

Evidence has shown that alkaloids, flavonoids, and phenylpropanoids are abundant in the *Meconopsis* species [9]. Additionally, the reports show that flavonoids are relatively higher than alkaloids in *M. integrifolia* alcohol extract [10], which could represent the functional constituents. At the same time, there are lots of reports demonstrating that flavonoids can induce cancer cell programmed death, which is called apoptosis [18,19]. For example, Kaempferol has recently been reported to suppress proliferation, induce cell cycle arrest and promote apoptosis in various human cancer cell lines. Diosmin was found to be the most potent genotoxic agent in DU145 cells, which resulted in its pro-apoptotic activity [18,19]. Based on these observations, we found that *M. integrifolia* could significantly inhibit K562 cell viability, and we speculated that the cell death caused by *M. integrifolia* could be apoptosis. To confirm this hypothesis, Annexin V/FITC was applied to evaluate the apoptosis ratio induced by the extract. As expected, the results (Figure 3A) indicated that *M. integrifolia* could induce K562 cell apoptosis. The apoptosis ratio increased from 23.4% to 67.8% accompanied by the increase of *M. integrifolia* from 30 to 90 μg/mL, while the control was 6.15%. A similar trend was observed after treatment for 48 and 72 h. The apoptosis ratio was in accordance with the cell mortality obtained from MTT assay, suggesting that *M. integrifolia* might primarily trigger K562 cell apoptosis. Membrane blebbing, cellular shrinkage, chromatin condensation and formation of apoptotic bodies are always the main morphological characteristics of apoptosis [20,21]. Therefore, we performed HO (Hoechst 33342) staining to further confirm the apoptosis induced by *M. integrifolia*. Data in Figure 3B clearly shows that *M. integrifolia* extract induced several features of apoptosis, such as condensed chromatin and fragmented punctate blue nuclear fluorescence in K562 cells. Caspase-3, one of the key executioners of apoptosis, is responsible for the proteolytic cleavage of many key proteins, such as the nuclear enzyme poly (ADP-ribose) polymerase (PARP) [22]. Herein, our results (Figure 3C) showed cleaved-Caspase 3 and cleaved-PARP expression was up-regulated after *M. integrifolia* extract incubation. Taken together, *M. integrifolia*-induced apoptosis might initiate in a caspase-dependent manner.

### 2.4. Mitochondria Play a Key Role Following M. integrifolia Incubation

**Figure 4:**
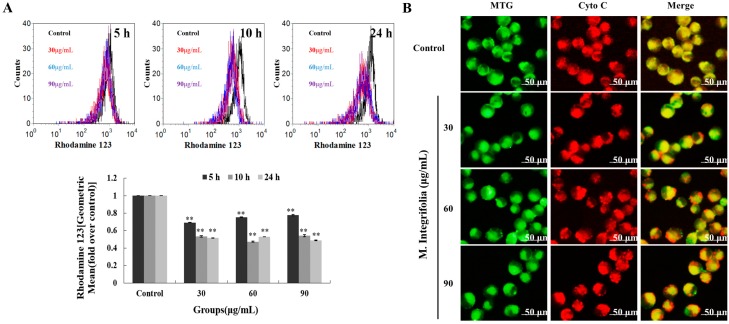
The alterations of mitochondria triggered by *M. integrifolia.* (**A**) Effect of *M. integrifolia* on mitochondrial membrane potential of K562 cells. Cells were treated with *M. integrifolia* for 5, 10 and 24 h. Then the cells were labeled with Rhodamine 123 and analyzed by flow cytometry. Histograms show number of cell channel (vertical axis) *vs.* Rhodamine 123 fluorescence (horizontal axis). ** *p* < 0.01 *vs.* Control. (Error bars = S.D., *n* = 3); (**B**) Detection of release of cytochrome C from mitochondria in K562 cells after *M. integrifolia* treatment.

Mitochondria are thought to be the major pathway for apoptosis, and, therefore, targeting them is a novel strategy for cancer therapy [23,24]. Mitochondria mediated apoptosis is highly regulated by the balance between the expression of pro- and anti-apoptotic proteins in the Bcl-2 family proteins [25,26]. If this balance is broken, the direct consequence is a loss of the mitochondrial transmembrane potential (Δψm) [27,28]. Thus, MMP (mitochondria membrane potential) loss was first analyzed. As shown in Figure 4A, significant MMP loss was detected after *M. integrifolia* incubation. The reduction of membrane potential leads to the release of mitochondrial cytochrome C, which is the key factor that results in the formation of apoptosomes. Our investigation revealed that *M. integrifolia* could significantly induce the release of mitochondrial cytochrome C (Figure 4B), which was in accordance with the results above. In addition, Caspase-9, another key factor involved in the mitochondria-mediated apoptosis, is always activated by cytochrome C. In our study, the protein activated the effector Caspase-3 and subsequently increased the cleavage of PARP expression and, finally, induced cell apoptosis. Western blot analysis (Figure 3C) also showed that Caspase-9/3 and PARP were all involved in *M. integrifolia* induced apoptosis in K562 cells. These results clearly indicated that *M. integrifolia* induced apoptosis via mitochondrial pathways.

### 2.5. M. integrifolia Initiates ROS Accumulation in K562 Cells

**Figure 5:**
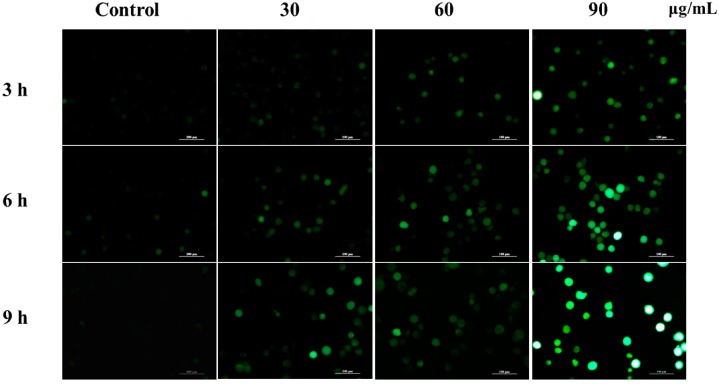
ROS generation induced by *M. integrifolia*. K562 cells were treated with *M. intergrifolia* for 3, 6 and 9 h. The intracellular ROS level was observed under fluorescent microscope.

ROS plays an important role in the apoptosis investigation and is mainly produced in mitochondria. Mounting evidence proved that the increasing ratio of Bax and Bcl-2 could decrease MMP, induce the release of cytochrome C, and eventually result in ROS accumulation. However, excessive amounts of ROS can cause oxidative damage to lipids, proteins, and DNA leading to tumorigenesis or cell death. To address the role that ROS played in *M. integrifolia* extract-induced apoptosis, ROS level was analyzed. In our experiment, *M. integrifolia* could significantly elevate ROS level in K562 cells, which indicated that ROS might have a function in the apoptosis process (Figure 5).

## 3. Experimental Section

### 3.1. Preparation for the M. integrifolia Extract

Fresh *M. integrifolia* plants were collected in Yushu County, Tibetan Autonomous Prefecture of Yushu Qinghai Province in August 2011 (altitude 4639 m). The plant species identities were authenticated by Associate Prof Jun-Hua Du at the School of Life and Geographical Sciences, Qinghai Normal University.

Dried *M. integrifolia* powder was homogenized in 95% ethanol at 45 °C using ultrasonic-assisted extraction (UAE) three times. The ethanol extract was obtained by vacuum filter, and the supernatant was collected and concentrated with a rotary evaporator (RE-2000 A; Belong, Shanghai, China). The ethanol fraction was homogenized in 75% ethanol and the supernatant was filtered with 0.22 μm filters.

### 3.2. Cell Cultures

K562 cells were obtained from the Institute of Chinese Academy of Medical Sciences, Beijing, China. K562 cells were cultured in RPMI-1640 medium containing 10% fetal bovine serum (FBS), 1% penicillin–streptomycin (100 U/mL penicillin and 100 μg/mL streptomycin) and 1% glutamine in cell culture flasks under a humidified 5% CO_2_ and 95% air atmosphere at 37 °C.

### 3.3. Cell Viability Tests

The cytotoxicity of *M. integrifolia* extract was evaluated by MTT (Sigma, St. Louis, CA, USA) assay. Briefly, 100 µL of K562 cells suspension was placed in 96-well culture plates at densities of 1 × 10^5^ cells/mL, and incubated with various concentrations of *M. integrifolia* extract (0, 10, 30, 60, 90, 120 and 150 µg/mL) for various time points (24, 48 and 72 h). Then, 10 μL MTT solution (5 mg/mL in phosphate buffer) was added into the cell suspension and the mixture was incubated for 4 h at 37 °C in a CO_2_ incubator. The formazan crystals were dissolved in 100 μL 10% SDS, 5% isobutene and 0.01 M HCL solution. Finally, the absorbance at 570 nm was recorded using a microplate reader (ELX800; BIO-TEK Instruments, Inc., Winooski, VT, USA). Cell survival was calculated using the following equation:

Cell survival (%) = OD treatment group/OD control group × 100%
(1)

The peripheral blood mononuclear cells (PBMCs) were isolated with the lymphocyte separation kit (Applygen Technologies Inc., Beijing, China) from healthy donors in the hospital of Shaanxi Normal University, China. The whole procedures were performed according to the instructions provided by the manufacturer. In this experiment, PBMCs were used as normal cells to evaluate the cytotoxicity of *M. integrifolia*. Briefly, K562 cells and PBMCs suspension were seeded at a density of 1 × 10^5^ cells/mL in 24-well culture plates, respectively, and incubated with *M. integrifolia* at different doses (0, 30, 60, 90, and 120 µg/mL) for 24 h. Two types of cells were then stained with trypan blue and counted under the microscope (Nikon Instech Co., Ltd., Tokyo, Japan). The cell viability was calculated using the following equation:

Cell viability (%) = number of unstained cells/total cell number × 100%
(2)

### 3.4. Phase-Contrast Microscopy Observation

Cells receiving different treatments were observed by the phase-contrast microscopy (Nikon) at a 400× magnification to study the cell number. Representative micrographs were taken using NIS-Elements software.

### 3.5. Apoptosis Detection by Flow Cytometry

Apoptotic cells were quantified with an Annexin V–FITC Apoptosis Detection Kit (Invitrogen, Carlsbad, CA, USA) according to manufacturer’s protocol. Briefly, cells at 1 × 10^5^ cells/mL were incubated with various concentrations (0, 30, 60 and 90 µg/mL) of *M. integrifolia* extract for 24, 48 and 72 h at 37 °C. Cells were then harvested and re-suspended in the binding buffer then stained with 2 µL Annexin V–FITC and 2 µL PI (propidium iodide) for 15 min at room temperature in the dark. The apoptotic index was immediately determined by flow cytometry.

### 3.6. Hoechst 33342 Staining

To detect changes in nuclei morphology of K562 cells after *M. integrifolia* extract treatment, Hoechst 33342 staining was performed. After treatment with the indicated concentration (0, 30, 60 and 90 μg/mL) of *M. integrifolia* extract for 24, 48 and 72 h, cells were stained by 10 µM HO for 15 min at room temperature. Then, the stained cells were washed three times with phosphate-buffered saline (PBS) and observed using a fluorescence microscopy with standard excitation filters (Nikon). The excitation wavelength was 346 nm and the emission wavelength was 460 nm.

### 3.7. Cell Cycle Analysis

The ratio of cells in the G0/G1, S and G2/M phases of cell cycle was determined by examining their DNA content. Cells at 1 × 10^5^ cells/mL were treated with various concentrations (0, 30, 60 and 90 μg/mL) of *M. integrifolia* extract for 24 h. Then, cells were harvested and washed twice with cold PBS and fixed with 70% ice-cold ethanol at 4 °C overnight. The fixed cells were washed twice with cold PBS and incubated with 100 µg/mL RNase A (Sigma) for 30 min at 37 °C. Cell were then stained with 50 µg/mL propidium iodide (PI; Sigma) for 30 min in the dark and analyzed by flow cytometry (Millipore, Bedford, MA, USA).

### 3.8. DNA Fragmentation Assay

To analyze DNA fragmentation, flow fluorocytometric detection of DNA hypoploidy after adding PI to the dying cells for permeabilization was performed [29]. The size of DNA fragments appeared as a hypoploid DNA histogram. To investigate the effect of *M. integrifolia* extract on DNA damage of K562 cells, oligonucleosomal DNA fragmentation by flow cytometry was performed. Cells were treated with various concentrations of *M. integrifolia* extract (0, 30, 60 and 90 μg/mL) for 24, 48 and 72 h, respectively, and then were stained with 5 µg/mL PI and analyzed for DNA content by flow cytometry.

### 3.9. Intracellular Reactive Oxygen Species Detection

In this study, we also evaluated changes in the cellular reactive oxygen species (ROS) level through the oxidative conversion of the sensitive fluorescent probe 2′,7′-dichlorofluorescein-diacetate (DCFH-DA) to fluorescent 2′,7′-dichlorofluorescein (DCF). K562 cells were treated with the mentioned concentrations (0, 30, 60 and 90 μg/mL) of *M. integrifolia* extract for 3, 6 and 9 h. The treated cells were harvested, washed twice with PBS, re-suspended in 500 µL of 10 µM DCFH-DA (purchased from Molecular Probes Inc., Invitrogen) and incubated at 37 °C for 30 min in the dark. The samples were observed using a fluorescence microscopy with standard excitation filters (Nikon).

### 3.10. Measurement of Mitochondrial Membrane Potential (MMP)

The alterations of MMP were assessed with the fluorescent probe Rhodamine 123 (Rh 123, Sigma). Rh123 selectively enters mitochondria with an intact membrane potential and is retained in the mitochondria. Once the MMP is lost, Rh123 is subsequently washed out of the cells. K526 cells (1 × 10^5^ cells/mL) were incubated with various concentrations of *M. integrifolia* for 5, 10 and 24 h. Then, the treated cells were stained with 2 μg/mL Rh123 for 20 min at 37 °C. After being washed with PBS, cells were immediately analyzed by flow cytometry.

### 3.11. Cytochrome C Detection

Cells in each treatment group were incubated with 0.5 mM Mito-tracker Green (MTG) in phosphate-buffered saline at 37 °C for 20 min, fixed in 4% paraformaldehyde at 4 °C for 15 min, and subsequently permeabilized with 0.1% Triton X-100 at 4 °C for 5 min. Between each step, cells were washed with PBS. After permeation, cells were blocked with 5% BSA at 37 °C for 1 h and then incubated with anti-cytochrome C antibody (Santa Cruz Biotechnology, Santa Cruz, CA, USA) overnight at 4 °C. After being washed with phosphate-buffered saline, the samples were incubated with fluorescein isothiocy-anate-conjugated secondary antibody. Finally, the cells were observed by fluorescence microscope (Nikon E 600).

### 3.12. Western Blotting Staining

SDS–PAGE and immunoblotting were performed according to standard procedures. Briefly, after treatment with *M. integrifolia* (0, 30, 60 and 90 μg/mL) for 24 h, K562 cells were lysed by RIPA buffer on ice. The protein samples were separated on a 10% SDS polyacrylamide gel, and the gel was transferred to nitrocellulose membranes (Millipore) and blotted with primary antibodies (Caspase-3, cleaved-Casepase-9, cleaved-PARP were purchased from Cell Signaling Technology, Danvers, MA, USA) overnight at 4 °C. The bound primary antibodies were then tagged with IRDye 680-conjugated IgG (Li-Cor Biosciences, Lincoln, NE, USA) at room temperature for 1 h. The infrared fluorescence was detected with the Odyssey infrared imaging system (Li-Cor Bioscience).

### 3.13. Statistical Analysis

All experiments were performed in triplicate, and data were expressed as the means ± SD. IC50 values were calculated by regression analysis. The data were subjected to an analysis of Duncan’s multiple range tests (SPSS, version 18.0). A significant difference was judged to exist at a level of * *p* < 0.05 and ** *p* < 0.01.

## 4. Conclusions

In conclusion, we first investigated the anticancer effect of *M. integrifolia* extract, which can block cell cycle processes and induce cell apoptosis in K562 cells. *M. integrifolia* extract induced-apoptosis might be mediated by the release of cytochrome C, ROS accumulation, and activation of Caspase-3 and Caspase-9. This study could provide the primary proof needed to understand this new function of *M. integrifolia.* As we know, natural products are increasingly popular due to their potential to improve the efficacy of conventional chemotherapy, thus, in the future, we will try to investigate the combination effect of *M. integrifolia* with chemotherapy drugs, hoping to apply it in a clinical setting.
